# *In vivo* Therapeutic Effects and Mechanisms of Hydroxyasiaticoside Combined With Praziquantel in the Treatment of Schistosomiasis Induced Hepatic Fibrosis

**DOI:** 10.3389/fbioe.2020.613784

**Published:** 2021-01-22

**Authors:** Huilong Fang, Ling Yu, Da You, Nan Peng, Wanbei Guo, Junjie Wang, Xing Zhang

**Affiliations:** ^1^Department of Pharmacology, Xiangnan University, Chenzhou, China; ^2^Affiliated Hospital of Xiangnan University, Chenzhou, China; ^3^Department of Trauma and Reconstructive Surgery, Rheinisch-Westfälische Technische Hochschule Aachen University Hospital, Aachen, Germany

**Keywords:** praziquantel, madecassoside, *Schistosoma japonicum*, liver fibrosis, pro-inflammatory factors

## Abstract

Schistosomiasis has been a fatal obstinate disease that threatens global human health, resulting in the granulomatous inflammation and liver fibrosis.

**Objective:**The aim of this study was to evaluate the therapeutic effects and mechanisms of hydroxyasiaticoside combined with praziquantel in the treatment of schistosomiasis-induced liver fibrosis.

**Methods:**Mice were randomly distributed into four experimental groups: normal control group, model group, praziquantel group, praziquantel + hydroxyasiaticoside group. Except for the normal control group, they were infected with *Schistosomia cercariae* through the abdominal skin to induce liver fibrosis. In the intervention group, mice were administered with the respective drugs by gavage after 8 weeks of infection. At the end of the treatment, mice were sacrificed to collect blood for the determination of aspartate aminotransferase (AST) and alanine aminotransferase (ALT) serum levels. Moreover, the liver was excised, weighed, and liver indices were calculated. Histopathological examination was performed to assess liver morphology. Besides, the expression of collagen type I and III in liver was determined; the mRNA expression levels of IL-6 and TNF-α in liver tissues were measured using Real-time PCR while ELISA and western blotting were performed on liver tissue homogenate to determine the protein expression of IL-6 and TNF-α.

**Results:**The combination of praziquantel and hydroxyasiaticoside lowered the pathological scores of schistosomiasis-induced hepatic fibrosis, the liver indice, serum AST and ALT levels, improved liver morphology, downregulated the expression levels of hepatic type I and III collagen, inhibited the mRNA expression levels of pro-inflammatory factors (IL-6 and TNF-α) in the liver of mice relative to the praziquantel alone.

**Conclusion:**The combination of hydroxyasiaticoside and praziquantel is a potential therapeutic option for schistosomiasis-induced hepatic fibrosis. Notably, this combination noticeably suppresses the protein and mRNA expression levels of pro-inflammatory factors (TNF-α and IL-6) in the liver.

## Introduction

In terms of public health impact, schistosomiasis is the second most important parasitic disease in the world (Tucker et al., 2013). Every year, 120 million symptomatic (Chitsulo et al., 2004), 70 million disability-adjusted (King et al., 2005) and schistosomiasis preferentially occurs in developing countries (Steinmann et al., 2006). Liver fibrosis is a wound-healing process that is aimed at restoring organ integrity after mechanical stress, autoimmune reactions and infections induced severe injury. The solicitation of this process is pathogenic and a pathognomonic feature of diseases like schistosomiasis (Campana and Iredale, 2017; Aydin and Akçali, 2018; Parola and Pinzani, 2019). Currently, there are still no efficacious therapeutic options for schistosomiasis-induced liver fibrosis.

In the past decades, traditional Chinese medicine has attracted significant attention due to its broad-spectrum anti-inflammatory and anti-fibrosis activities (Zhou et al., 2016; Yao et al., 2019). *Centella asiatica* is the whole dry grass of *C. asiatica* of umbelliferae used for clearing heat, dampness, detoxification and detumescence (Bylka et al., 2014). Hydroxyasiaticoside is a triterpenoid saponin that is isolated from *C. asiatica*. It has been reported that hydroxyasiaticoside has numerous significant biological effects, such as anti-inflammation, anti-oxidation, anti-depression, anti-tumor, and anti-scar proliferation (Fitri et al., 2018a,b; Song et al., 2018; He et al., 2019). However, it has not been established whether hydroxy asiaticoside can alleviate schistosomiasis-induced fibrosis.

To further demonstrate our hypothesis, in our study, we successfully established a mice model of schistosomiasis-induced liver fibrosis that were utilized to assess the therapeutic efficacies and mechanisms of a combination of hydroxyasiaticoside and praziquantel. Results have shown that the combination of hydroxyasiaticoside and praziquantel not only consistently improved the liver fibrosis extent and lowered the pathological scores but also efficaciously reversed the rising level of AST and ALT released by the damaged liver cells. Impressively, this combination also remarkably inhibited the pre-inflammatory mediators release of IL-6 and TNF-α, which provides a specific target in the screening of anti-inflammatory drugs for the treatment of schistosomiasis-induced liver fibrosis. Herein, we effectively evaluate the significant roles of a combination of hydroxyasiaticoside and praziquantel, which open up new avenues for exploring alternative therapeutic options in treating schistosomiasis-associated liver fibrosis.

## Materials and Methods

### Experimental Animal

Eighty male Kunming mice weighing about 18–22 g were purchased from Dongchuang Experimental Animal Science and Technology Service Department. The mice were allowed 3 days to acclimatize to the environment before experimentation. Mice were freely provided with drinking water and standard pellet feeds. Positive snails were purchased from Hunan Institute of Schistosomiasis Control. Cercariae were acquired by routine methods at 24–28°C in the laboratory.

### Drugs and Materials

*Centella asiatica* with standard purity of 98.93% (HPLC), batch number: MUST-19032915, were purchased from Chengdu Manster Biotechnology Co., Ltd. The chromatographic conditions used were as follows: Shimadzu InertSustain C18 column (4.6 × 250 mm, 5 μm); Mobile phase: acetonitrile: methanol: water=26: 24: 50; Flow rate: 0.8 ml/min; Detection wavelength: 204 nm; Injection volume: 10 μL. Praziquantel was purchased from Nanjing Pharmaceutical Co., Ltd. Aspartate aminotransferase (AST), alanine aminotransferase (ALT) kits, and other regents were obtained from Nanjing Jiancheng Biotechnology Co., Ltd.

### Experimental Method

#### Animal Model Preparation and Experimental Set-Up

Mice were randomly distributed into four groups: normal control group, model group, praziquantel group, and praziquantel + hydroxyasiaticoside group. To successfully establish mice models of schistosomiasis-induced fibrosis, mice were fixed to a plate, and their hair on a small skin area of the lower abdomen was shaved. Subsequently, the exposed naked skin was rinsed with normal saline twice. Twenty five cercariae were microscopically counted on the cover slides. Except for the normal control group, the cover slides were then applied to the exposed skin of mice for about 10 min. Appearance of several hyperemic spots on the skin indicated the successful cercariae infection.

#### Experimental Animal Administration

Mice in the normal control group were not infected with the cercariae of *Schistosoma japonicum*. For the experimental control group, mice infected with the cercariae were intragastrically administered with 0.5 mL of normal saline each day for consecutive 8 weeks. Mice in the praziquantel group were intragastrically administered with praziquantel at a dose of 500 mg/kg (0.5 mL) for consecutive 2 days after the cercariae infection, followed by the administration of 0.5 mL of saline. For the praziquantel + hydroxyasiaticoside group, mice after the cercariae infection were intragastrically administered with praziquantel at the equal dose for 2 days, followed by hydroxyasiaticoside administration with a dose of 0.5 mL of 40 mg/kg for consecutive 8 weeks. At the end 16th week, mice were fasted for 16 h and then weighed. Finally, mice in different groups were anesthetized, blood samples were then collected from eyeballs for further tests.

#### Liver Index Calculation and Histochemical Staining

The obtained blood was centrifuged at 3,000 rpms for 10 min after which serum was obtained. Serum ALT and AST levels were determined using ELISA kits. Subsequently, mice were sacrificed by cervical dislocation. Their livers were excised, and weighed to calculate the liver indices (the number of milligrams of liver per gram body weight). The middle lobe of the liver tissue was fixed in 4% paraformaldehyde and then gradient-dehydrated *via* various concentrations of ethanol. These samples were embedded in liquid paraffin (Zhang et al., 2019b). It was then sectioned, stained with H&E and Masson reagents, and cover slipped. Pathological changes of the liver tissue were visualized and observed under a light microscope. The degree of liver fibrosis was divided into 4 grades (Parola and Pinzani, 2019): Grade 0 (20.1 points)–normal; grade I (21.2 points)–collagen fibers wrapped around the granuloma and inserted into it; grade II (22.2 points)–the existence of intense fibrosis in the portal area and only a small amount of fibrosis between lobules; grade III (2308 points)–a large amount of fibrous tissue extending into the interlobular. Moreover, the type I and III collagen levels in the liver were also determined. Briefly, the immunohistochemically stained sections of the liver were observed under *20 visual field of objective lens. Twenty visual fields were randomly selected in each group, and the average absorbance of brown granules in cells were measured by automatic color image analysis system.

#### ELISA and Western Blot Analysis of Protein Levels of TNF-α and IL-6

In the ELISA experiments, the known antigen was firstly diluted to 1–10 μg/ml with coating buffer and add 0.1 ml of diluted antigen solution to each well overnight. The samples were then washed thrice on the next day. Afterwards, we added 0.1 ml of the diluted sample to each reaction well and incubated for 1 h. Subsequently, 0.1 ml of freshly diluted enzyme-labeled secondary antibody was introduced and incubated at 37°C for 30 min. At last, 0.1 ml of the temporarily prepared TMB substrate solution and 0.05 ml of 2 M sulfuric acid was added to each reaction well and reacted for 30 min. The OD value was determined by enzyme labeling at 450 nm using an ELISA tester after coloring.

For western blot experiments, the BCA protein assay kit was used to determine the protein concentration of each sample. Equal amounts of protein samples were then scraped into SDS-PAGE gel electrophoresis, followed by electro-transfer to PVDF membrane. These membranes were incubated with primary antibodies at 4°C overnight and horseradish peroxidase-conjugated secondary antibodies for 2 h. The immune complexes of each group were detected by ECL WB detection system.

#### Real-Time Quantitative PCR Analysis

mRNA expression levels of samples in different groups were measured using Real-time PCR. Total RNA was isolated from liver tissues using a TRIzol Reagent (ThermoFisher, U.S.A). Approximately 1 ug of RNA was reverse-transcribed into cDNA, which was utilized as a template for real-time PCR detection *via* SYBR Premix Ex Taq reagent kit (TaKaRa, Japan). Expression levels were calculated using the 2^−Δ*CT*^ method. Primers used for RT-PCR analysis are shown following Table 1.

**Table 1:** The Sequences of the Primers.

**Gene name**	**Upstream sequence**	**Downstream sequence**
IL-6	TGATGCTGTTGCTGCTGCTGAG	CACATTCTGGAGGAAGTCCTTGGC
TNF-α	GCCTCTTCTCATTCCTGCTTGTGG	GTGGTTTGTGAGTGTGAGGGTCTG
β-actin	AGAGGGAAATCGTGCGTGAC	CAATAGTGATGACCTGGCCGT

### Statistical Processing

SPSS 21.0 software was utilized to analyze the experimental data. Mean differences between groups were compared with ANOVA and Student's test. All data are expressed as mean ± standard deviation. *p* ≤ *0.05* was set as the threshold for statistical significance.

## Results

### Characterization of *Centella asiatica* Extract

Hydroxyasiaticoside was extracted from *C. asiatica* by ethanol reflux method. Then it was separated and purified. Finally, it was characterized using a high-performance liquid chromatography (HPLC). The purity of hydroxyasiaticoside was found to be 90.9%. The required concentration was prepared by dilution in normal saline. The regression equation of the standard curve of hydroxyasiaticoside: *Y* = 282.96X-23.171, *R*^2^ = 0.9998, and the standard curve are shown in Supplementary Figure 1.

### Comparison of Liver Morphology and Liver Index

For mice in the normal control group, the liver surface appeared smooth, ruddy, soft, with sharp edges and an intact capsule. Additionally, no pathological features of liver fibrosis were observed. On the contrary, the livers of model group mice were dark-brown, enlarged, brittle, and accompanied with the tense capsule and even distribution of granular processes. Although liver appearances in the praziquantel and praziquantel + hydroxyasiaticoside groups were slightly different with compared to those of the normal control group, they were noticeably improved compared to the model group.

**Figure 1:**
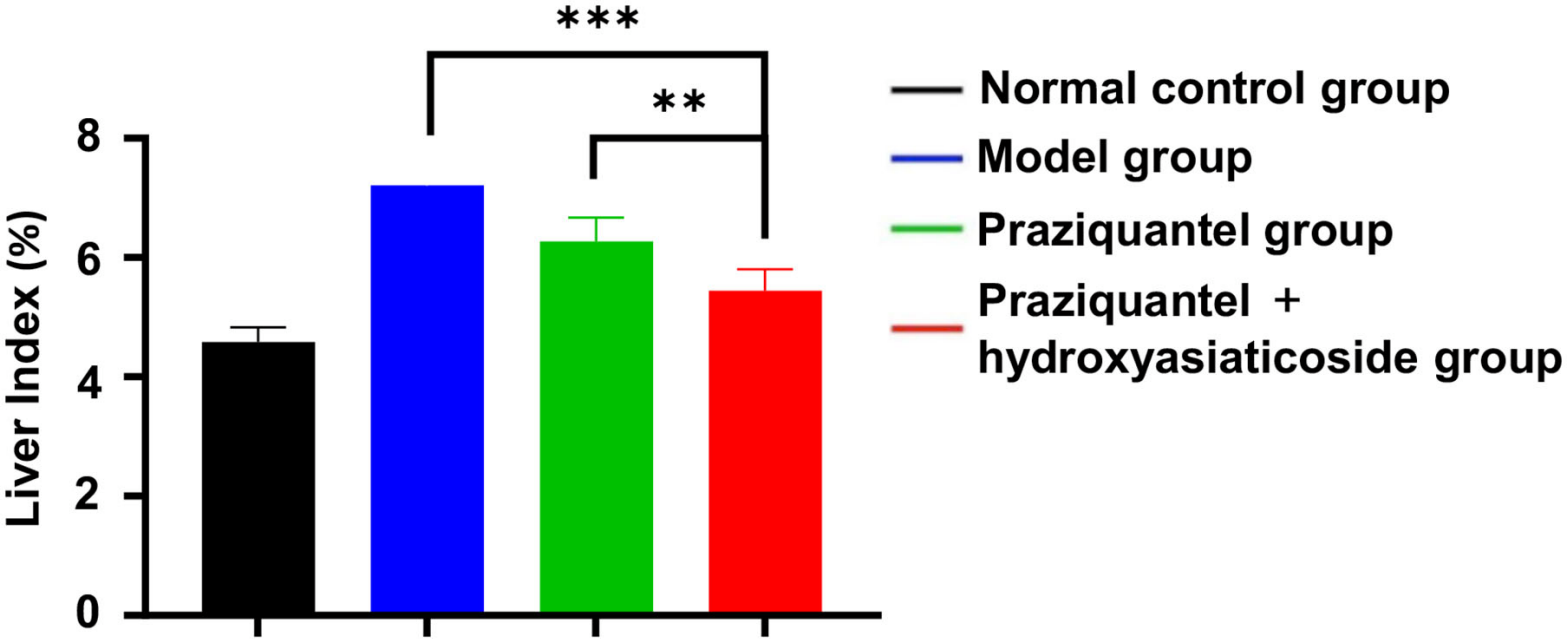
Comparison of liver index of mice in various treatment groups. **p < 0.01, ***p < 0.001.

Figure 1 shows that the liver index of model group was significantly higher than that of the normal control group. Furthermore, the praziquantel group exhibited a decrease in liver index when compared to the model group. This was attributed to a certain degree of attenuation effect from praziquantel treatment. Notably, praziquantel + hydroxyasiaticoside group exhibited the lowest liver index among all treatment groups, demonstrating that the combination of praziquantel and hydroxyasiaticoside exerted a desirable curative impact on the liver index.

### H and E and Masson Staining of Liver Tissues and Liver Fibrosis Scores

**Figure 2:**
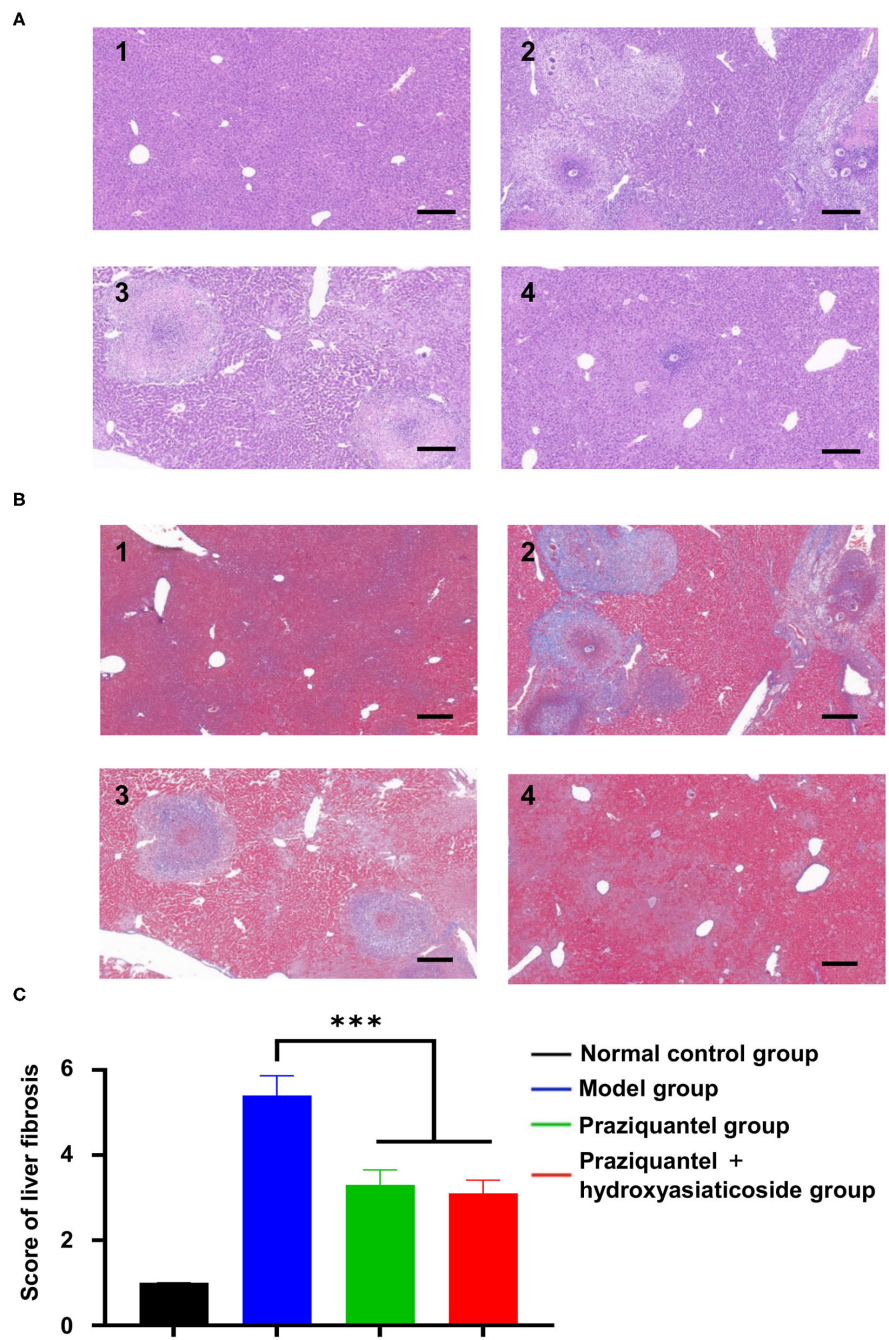
H&E **(A)** and Masson **(B)** staining observation of morphological changes of liver tissue sections of mice in different treatment groups. Scale bar: 200 μm. 1: normal control group; 2: model group; 3: praziquantel group; 4: praziquantel + hydroxyasiaticoside group. **(C)** Comparisons of the liver fibrosis scores in various groups. ***p < 0.001.

It is noteworthy that no significant liver pathological changes were observed in the normal control group. However, substantial chronic egg granulomas surrounded by spindle cells and collagen fibers were found in the model group, praziquantel group, and praziquantel + hydroxyasiaticoside treatment group, as shown in Figure 2. A small amount of acute egg granuloma and a large quantity of inflammatory cells were observed in the portal area, and the collagen fiber bundles around the granuloma and venules surrounded the hepatic lobules. As given in Figures 2A,B, the extent of liver tissue lesion in the praziquantel + hydroxyasiaticoside group was significantly low than that of the praziquantel group. This finding confirmed that a combination of praziquantel and hydroxyasiaticoside was more effective in ameliorating the lesion compared to praziquantel alone. The liver fibrosis scores in various treatment groups was calculated based on the grade standard of liver fibrosis. As shown in Figure 2C, the praziquantel group and praziquantel + hydroxyasiaticoside group exhibited noticeably lower hepatic fibrosis scores relative to the model group (*p* < 0.001). However, the difference in scores between these two treatment groups was not significant (*p* > 0.05).

### Serum of ALT and AST Levels

**Figure 3:**
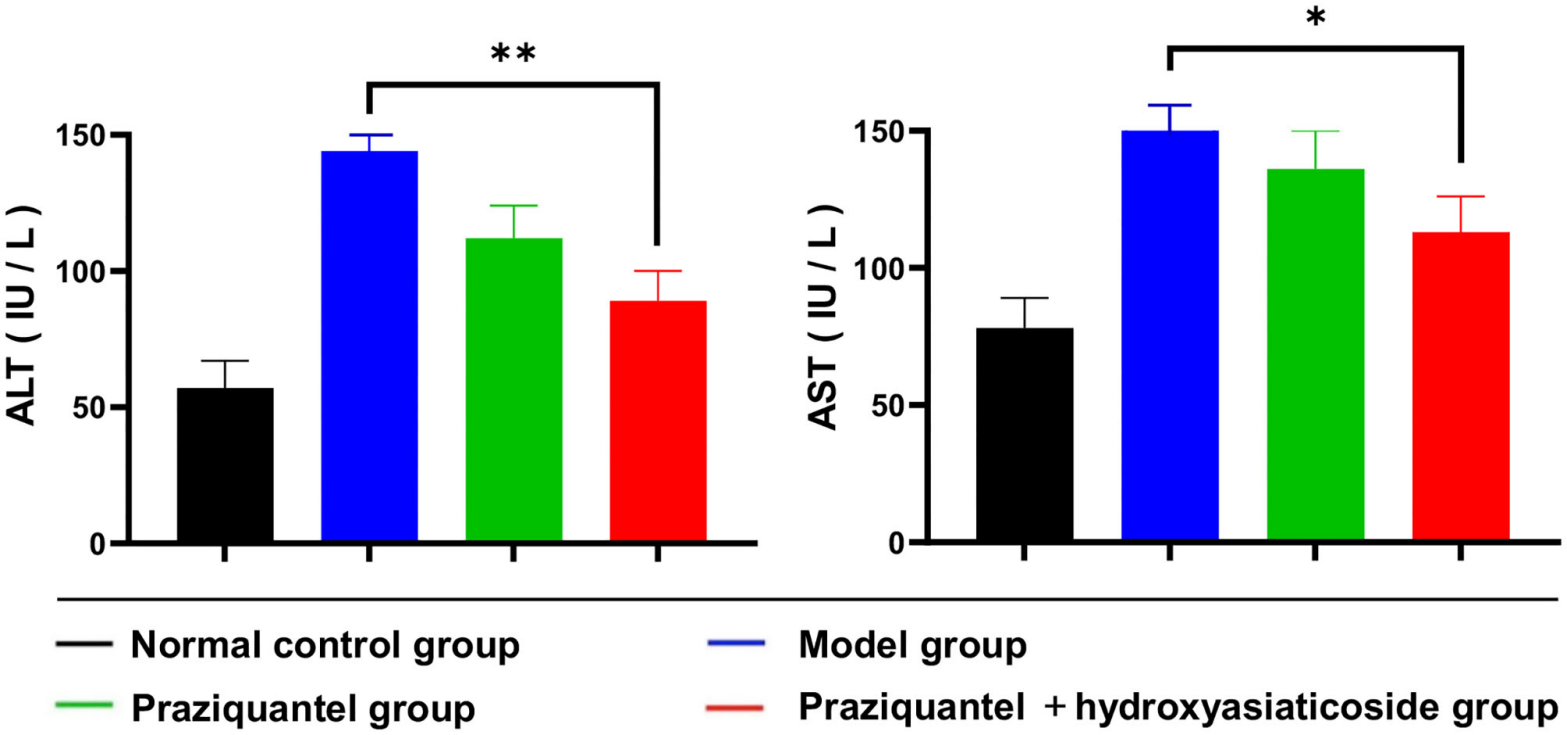
Comparisons of serum ALT and AST levels in mice. *p < 0.05, **p < 0.01.

*Schistosoma japonicum* infection elevated serum ALT and AST levels. Figure 3 shown that serum ALT and AST levels in the praziquantel group were remarkably low than those of the model group. Nevertheless, ALT and AST levels in the praziquantel + hydroxyasiaticoside group continued to decrease and remained relatively low when compared to the praziquantel group. This validated that a combination of praziquantel + hydroxyasiaticoside is an efficacious approach for ameliorating *S. japonicum*-induced elevated serum ALT and AST levels. The numerical variation of serum ALT and AST levels in the different treatment groups was as shown in Supplementary Table 1.

### Immunohistochemical Staining and Analysis of Type I and III Collagen Expression

**Figure 4:**
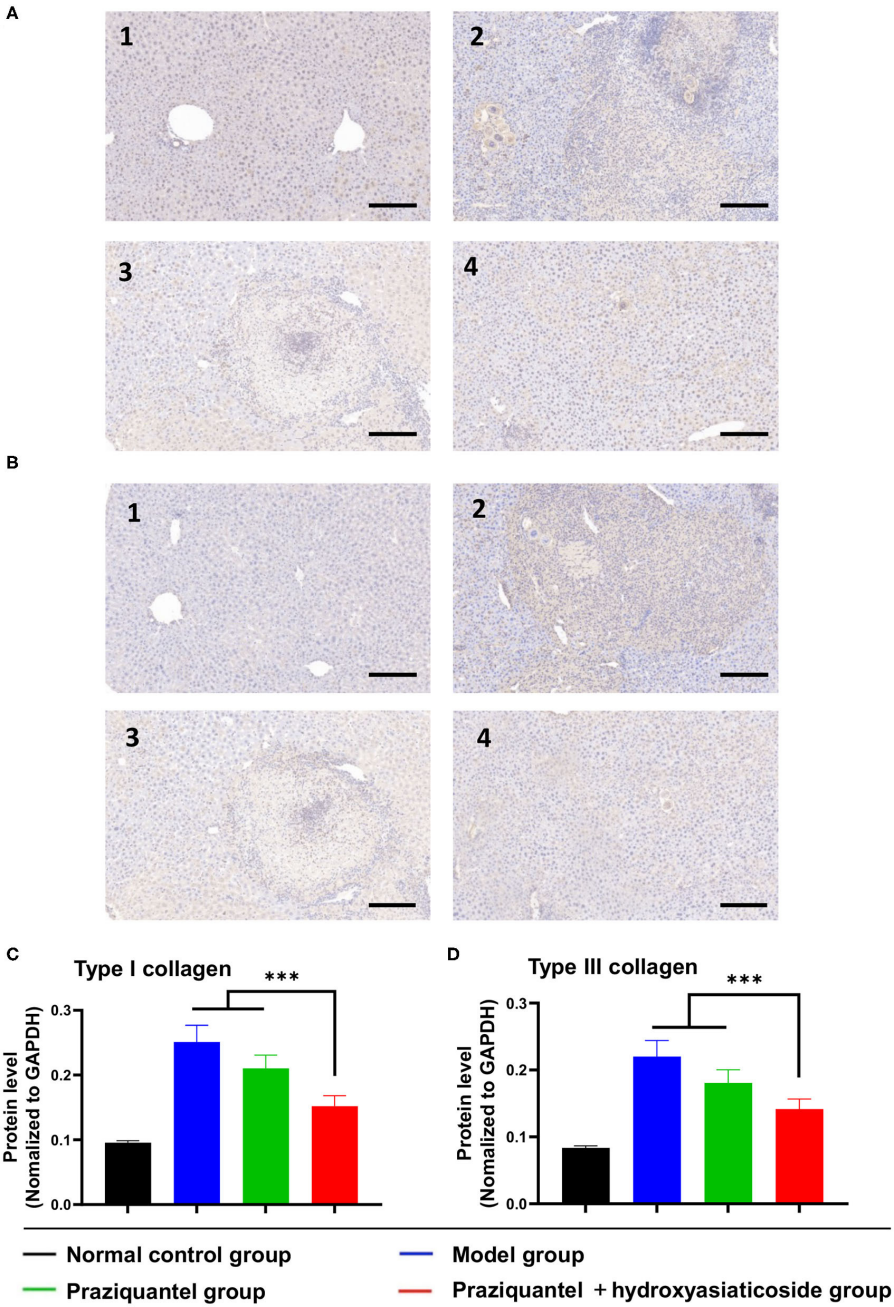
Immunohistochemistry analysis of the expression levels of type I **(A)** and III **(B)** collagen in liver tissues. Scale bar: 200 μm. Quantitative detection of the expression levels of type I **(C)** and III **(D)** collagen among different groups. 1: normal control group; 2: model group; 3: praziquantel group; 4: praziquantel + hydroxyasiaticoside group. ***p < 0.001.

Figures 4A,B displayed that hepatic type I and III collagen appeared brown, dense flaky, and were mainly distributed in egg granuloma and portal areas, especially in the model group. In the normal control group, there was no observable collagen staining. Besides, the brown hepatic type I and III collagen appeared to be improved to a certain extent after subjecting to the praziquantel or praziquantel + hydroxyasiaticoside treatments. As indicated in Figures 4C,D, the expression of type I and III collagen in the model group was noticeably higher than in the normal control group. Compared to either model group or praziquantel group, a combination of praziquantel and hydroxyasiaticoside given rise to significantly low hepatic type I and type III collagen levels in liver tissues (*p* < 0.001). This revealed that a combination of praziquantel and hydroxyasiaticoside had obvious superiorities in suppressing collagen levels compared to praziquantel alone. The numerical variation of hepatic type I and type III collagen levels in the various groups were shown in Supplementary Table 2.

### Protein Expression Levels of TNF-α and IL-6

**Figure 5:**
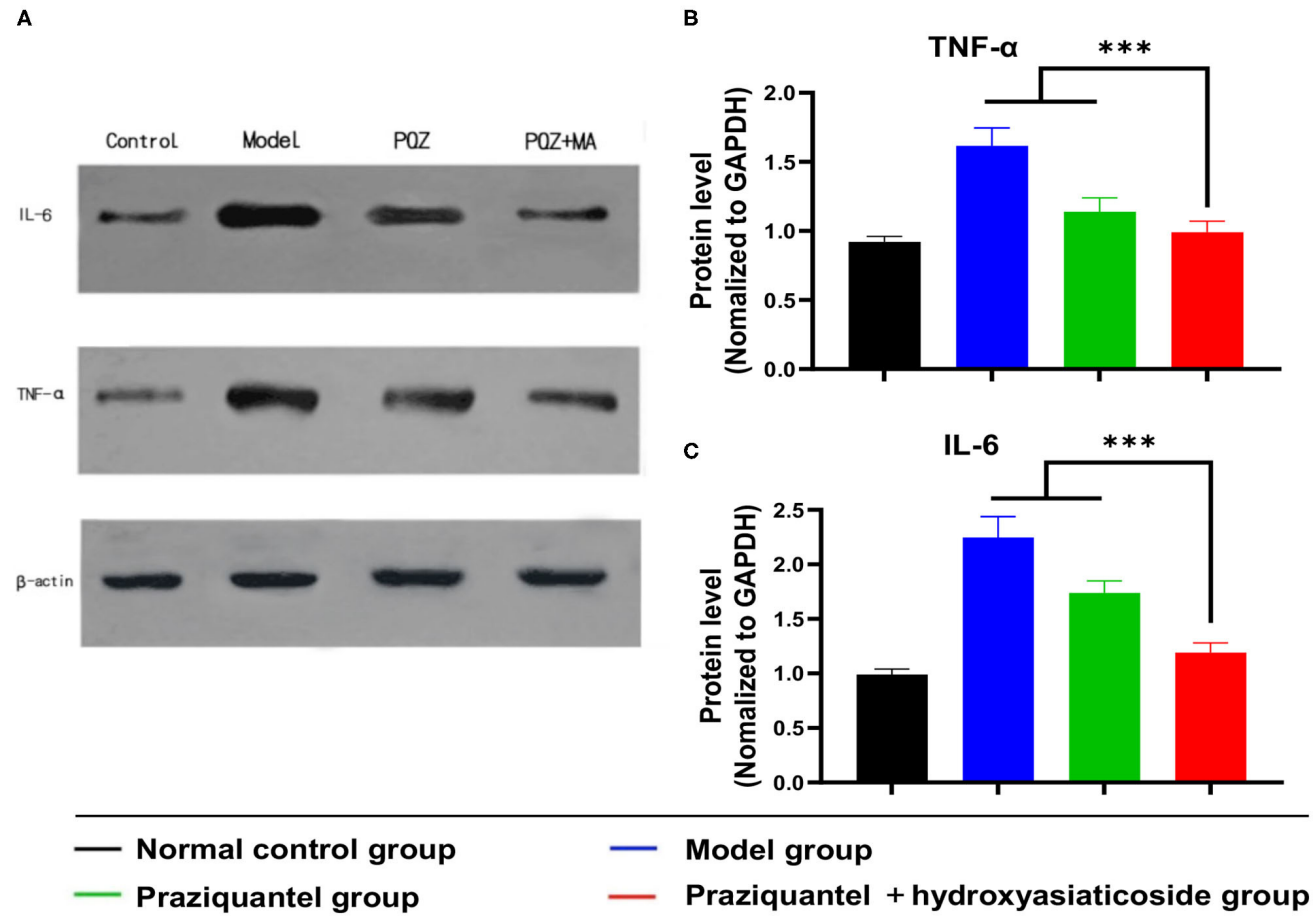
**(A)** Western blot analysis of protein levels of IL-6 and TNF-α in liver tissues. Quantitative ELISA detection analysis of protein levels of TNF-α **(B)** and IL-6 **(C)**. ***p < 0.001.

Cytokines, such as TNF-α and IL-6, play an essential role in the progression of the schistosomiasis. *Schistosoma japonicum* infection inevitably results in a noticeable elevation of the expression levels of TNF- α and IL-6 proteins in damaged liver tissues of mice. Mice in the model group exhibited high TNF-α and IL-6 protein levels owing to schistosomiasis-induced liver tissue damage (Figure 5A). Of note, the expression levels of TNF-α and IL-6 proteins in the praziquantel group was lower than those of the model group from western blotting observation. Notably, ELISA analysis shown that the praziquantel + hydroxyasiaticoside group exhibited the lowest expression levels of TNF-α and IL-6 (Figures 5B,C), revealing a favorable inhibition effect of praziquantel + hydroxyasiaticoside on the pro-inflammatory factors. They could be exploited as therapeutic drug combinations for inhibiting inflammation cytokines secretion, therefore, improving overall curative performance.

### mRNA Expression of TNF-α and IL-6 in Liver Tissues

**Figure 6:**
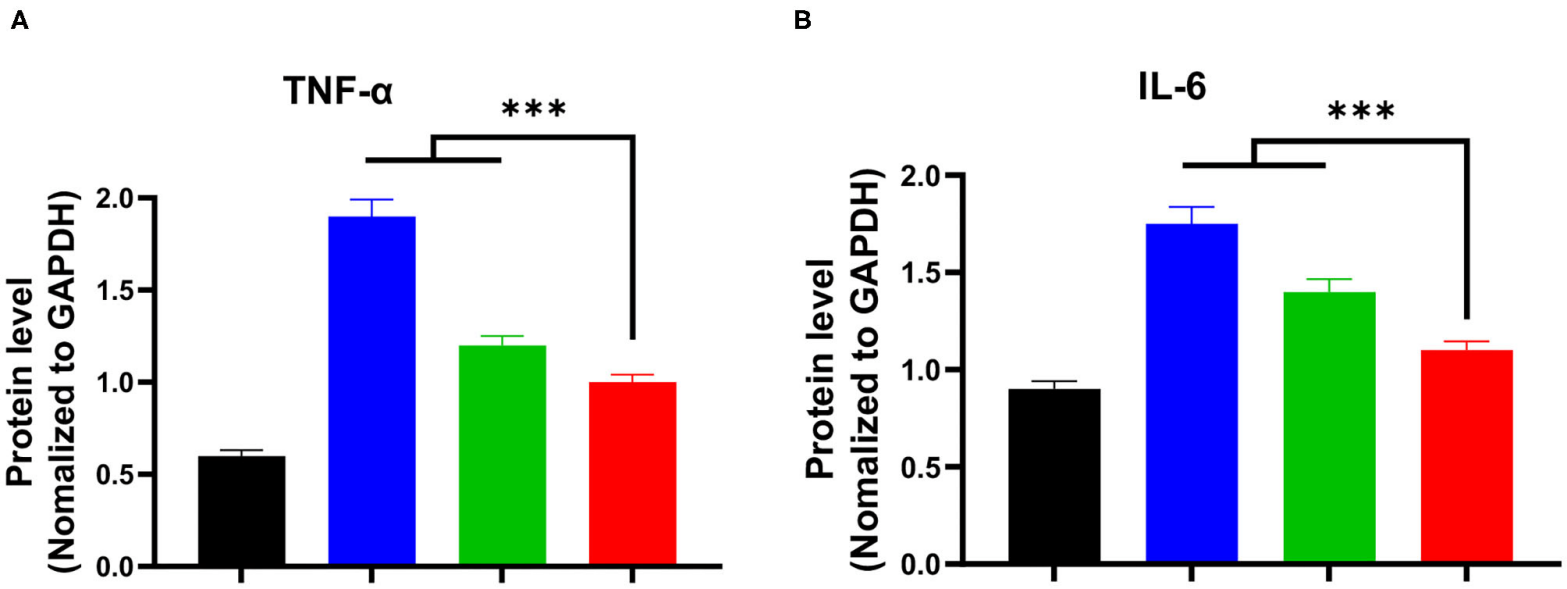
The mRNA expression levels of TNF-α **(A)** and IL-6 **(B)** in the liver among various groups. ***p < 0.001.

Afterwards, we further investigated the mRNA expression levels of TNF-α and IL-6 in liver tissues using RT-PCR, and the obtained results are presented in Figures 6A,B. It was found that the *S. japonicum* infection remarkably elevated mRNA expression levels of pro-inflammatory factors (TNF-α and IL-6) in the model group. Compared to the model group, the praziquantel group exhibited a certain inhibitory effect on the mRNA expression levels of TNF-α and IL-6. Impressively, there was a lowest mRNA expression levels of TNF-α and IL-6 in praziquantel + hydroxyasiaticoside group, which was slightly higher than the normal control group. This clearly demonstrated the satisfactory inhibitory effect on the mRNA expression of pro-inflammatory factors.

## Discussion

Schistosomiasis is a fatal disease that threatens human health, and more than 150 million people are infected with schistosomiasis globally (Bylka et al., 2014; Zhou et al., 2016; Fitri et al., 2018a). Previous studies showed that liver fibrosis symptoms due to *S. japonicum* can persist for a long time after complete disinfestation (Fitri et al., 2018b; Song et al., 2018; He et al., 2019). Patients with advanced schistosomiasis-induced liver fibrosis gradually develop cirrhosis due to egg deposition, and often die of gastrointestinal bleeding and liver failure caused by portal hypertension (Barnett, 2018; Chuah et al., 2019; Lewis and Tucker, 2019). Numerous studies have documented that liver fibrosis can be reversed, but liver cirrhosis is totally irreversible (Kamdem et al., 2018; Cai et al., 2019; Liu et al., 2019). Therefore, it is of importance to develop strategies for preventing or treating liver fibrosis.

Hydroxyasiaticoside has anti-inflammatory, antioxidant, and anti-tumor effects (Filgueira et al., 2018; Chen et al., 2019). However, it had not been established whether hydroxyasiaticoside can improve *S. japonicum* induced liver fibrosis. In this study, we found that 16 weeks after infection with the cercariae of *S. japonicum*, mice developed morphological and pathological features of liver fibrosis, and simultaneously, there was a significant decrease of liver indices. After 8 weeks of treatment with praziquantel, the extent of liver fibrosis in mice infected with cercariae was lower relative to the model group. It was noted that the liver fibrosis extent in mice after treatment with hydroxyasiaticoside for another 8 weeks consistently improved and relieved due to the role of hydroxyasiaticoside. This indicated that hydroxyasiaticoside is effective as a therapeutic option for early liver fibrosis-induced by schistosomiasis. It is well-acknowledged that the damaged liver cells could release some enzymes and other substances into the serum. In this study, we found that *S. japonicum* infection remarkably elevated serum ALT and AST levels. However, the treatment with hydroxyasiaticoside efficaciously lowered and reversed the rising ALT and AST expression levels. This clearly indicated that hydroxyasiaticoside had an effective hepatoprotective effects on liver cells.

Fibrosis is a self-repairing response after tissue damage. It is mediated by inflammation and persistent liver inflammation is a prerequisite for the formation of liver fibrosis (Zhou et al., 2014; Lurie et al., 2015; Campana and Iredale, 2017; Zhao et al., 2017b; Fernández-Ruiz and Aguado, 2018; Šibíková et al., 2018; Song et al., 2019). Abnormal cytokine production during inflammation can lead to the synthesis/degradation of the extracellular matrix (ECM) of liver. Cytokines such as TNF-α, IL-6, and IL-1 β, which are significant mediators of tissue damage, play an essential role in the progression of schistosomiasis (Qi et al., 2014; Yukinori and David, 2017; Jones and Jenkins, 2018; Li et al., 2019). TNF-α is induced by a wide range of pathogenic stimuli, and exerts a specific impact on the inflammatory response (Feng et al., 2019; Zhang et al., 2019a). IL-6 regulates neutrophil transport by coordinating chemokine production and leukocyte apoptosis (Ghasemi, 2018; Giudice and Gangestad, 2018; Tseng et al., 2019; Wang et al., 2020; Yu et al., 2020). Therefore, inhibition of pro-inflammatory mediators provides a specific target and direction in the screening of anti-inflammatory drugs. These results collectively indicate that hydroxyasiaticoside noticeably inhibits the expression levels of TNF-α and IL-6, and simultaneously improves schistosomiasis-induced liver fibrosis by regulating inflammatory response factors *in vivo*.

## Data Availability Statement

The original contributions presented in the study are included in the article/Supplementary Materials, further inquiries can be directed to the corresponding author/s.

## Ethics Statement

The animal study was reviewed and approved by Ethics Committee of Chenzhou University.

## Author Contributions

All authors wrote the manuscript and have given approval to the final version of the manuscript.

## Conflict of Interest

The authors declare that the research was conducted in the absence of any commercial or financial relationships that could be construed as a potential conflict of interest.
