# Supplementary material for: In vivo Therapeutic Effects and Mechanisms of Hydroxyasiaticoside Combined With Praziquantel in the Treatment of Schistosomiasis Induced Hepatic Fibrosis
Source: Front Bioeng Biotechnol. 2021 Jan 22;8:613784. doi: 10.3389/fbioe.2020.613784 (PMC7862569; doi:10.3389/fbioe.2020.613784)
Supplement: Supplementary file 1 [file Data_Sheet_1.docx]

***In vivo* therapeutic effects and mechanisms of hydroxyasiaticoside combined with praziquantel in the treatment of schistosomiasis induced hepatic fibrosis**

**Huilong Fang^1^，Ling Yu^2^，Da You^1^，Nan Peng^1^，Wanbei Guo^1^，Junjie Wang^1^* & Xing Zhang^3^***

1. Department of Pharmacology，Xiangnan University, Chenzhou 423000, P.R. China.
2. Affiliated Hospital of Xiangnan University, Chenzhou, 423000, P.R. China.
3. Department of Trauma and Reconstructive Surgery, Rheinisch-Westfälische Technische Hochschule Aachen University Hospital, Aachen 52074, Germany.

***Corresponding author**

Junjie Wang (*)

Department of Pharmacology，Xiangnan University,

Chenzhou, China, 423000

Email: [475911403@qq.com](mailto:475911403@qq.com)

Xing Zhang (*)

Department of Trauma and Reconstructive Surgery,

RWTH Aachen University Hospital,

Aachen 52074, Germany

Email: zhangxing0529dqwc@outlook.com

**Figure S1**. Standard curve of hydroxyasiaticoside.

**Table S1**

| Group | ALT(IU/L) | AST(IU/L) |
| --- | --- | --- |
| Normal control group Model group Praziquantel group Praziquantel + hydroxyasiaticoside group | 57±10  144±15  112±12  89±11 | 78±11  165±16  136±14  113±13 |

**Table S1**. Serum levels of ALT and AST in mice infected with schistosomiasis in various treatment groups（$\overline{x}$±s）

**Table S2**

| Group | Visual field number | Type Ⅰ collagen | Type Ⅲ collagen |
| --- | --- | --- | --- |
| Normal control group Model group Praziquantel group Praziquantel + hydroxyasiaticoside group | 20  20  20  20 | 0.0956±0.0031  0.2512±0.0258  0.2103±0.0207  0.1519±0.0161 | 0.0837±0.0030  0.2202±0.0239  0.1805±0.0199  0.1417±0.0148 |

**Table 2**. Immunohistochemical detection of expression level of type Ⅰ and Ⅲ collagen in liver tissues in different groups (average absorbance).
